# Modulation of the Hyperglycemia Condition in Diabetic Lab Rats with Extracts of the Creole Jamaica Flower (*Hibiscus sabdariffa* L.) from the Morelia Region (Mexico)

**DOI:** 10.3390/antiox13081010

**Published:** 2024-08-19

**Authors:** Teodoro Suárez-Diéguez, Marta Palma-Morales, Gloria Isabel Camacho Bernal, Erick Noe Valdez López, Celia Rodríguez-Pérez, Nelly del Socorro Cruz-Cansino, Juan Antonio Nieto

**Affiliations:** 1 Área Académica de Nutrición, Instituto de Ciencias de la Salud, Universidad Autónoma del Estado de Hidalgo, Abasolo 600, Colonia Centro, Pachuca de Soto E42000, Hidalgo, Mexico; tsuarez@uaeh.edu.mx (T.S.-D.); gloria_camacho11174@uaeh.edu.mx (G.I.C.B.); envl_9203@hotmail.com (E.N.V.L.) ncruz@uaeh.edu.mx (N.d.S.C.-C.); 2 Biomedical Research Centre, Institute of Nutrition and Food Technology (INYTA) José Mataix, University of Granada, Av. Del Conocimiento s/n, E18071 Granada, Spain; martapalm@ugr.es; 3 Department of Nutrition and Food Science, Faculty of Farmacy, University of Granada, Cartuja Campus, E18011 Granada, Spain; 4 Instituto de Investigación Biosanitaria de Granada (ibs.GRANADA), E18012 Granada, Spain; 5 Bioactivity and Nutritional Immunology Group (BIOINUT), Faculty of Health Science, Universidad Internacional de Valencia (VIU), Calle Pintor Sorolla 21, E46002 Valencia, Spain; janieto@universidadviu.com

**Keywords:** *Hibiscus sabdariffa*, bioactive compounds, antioxidant, phenolic compounds, diabetes, hyperglycemia

## Abstract

Extracts from Jamaica flowers (*Hibiscus sabdariffa*) from Morelia (Mexico) were evaluated as antidiabetic ingredients in a diabetic rat lab model for 80 days at doses of 200, 400, and 600 mg extract/kg rat weight. The hydroalcoholic extract (water:ethanol 80:20 (*v*/*v*) at 50 °C) showed a TPC value of 403.28 ± 7.71 mg GAE/g extract, and an antioxidant activity of 0.219 ± 0.00003 mmol Trolox/g (ABTS) and 0.134 ± 0.00001 mmol Trolox/g (DPPH). The extract allowed reducing the diabetic glucose plasma levels under fasting conditions in a dose-dependent manner by 35.2%, 41.63%, and 50.1%. Additionally, the highest dose of the extract (600 mg/kg) slightly reduced the short-term postprandial glucose response while improving the long-term response, reducing hyperglycemia by 45.1%. The same dose also improved lipid metabolism by reducing total cholesterol, triglycerides, VLDL, and LDL, while the HDL level increased. The improvement in glucose and lipid management in the treated groups also led to reduced levels of glycosylated hemoglobin, as well as lower insulin resistance (TyG index), compared to the diabetic control group. The results of this study suggest that extracts from *Hibiscus sabdariffa* (Morelia) can be used as potential functional ingredients or nutraceuticals for managing the diabetic condition.

## 1. Introduction

Diabetes mellitus is a metabolic disease characterized by a hyperglycemia condition because of the reduction in the number of islets’ β-cell numbers or defects in islet β-cell function [1]. Additionally, diabetes is associated with increased oxidative stress and, consequently, with other metabolic pathologies such as hypercholesterolemia, dyslipidemia, impaired protein metabolism, diabetic retinopathy, nephropathies, neuropathies, ulcerations, and inflammations [2]. Consequently, varied research has been conducted to elucidate the potential benefits of foods rich in phenolic compounds or phenolic extracts from various plant sources as glycemic modulators [3,4]. In that respect, it has been suggested that phenolic compounds may act as antidiabetic molecules by reducing or inhibiting the activity of α-amylase or α-glycosidase enzymes [5,6,7], acting as glucose competitors during intestinal absorption [8], modulating the genetic expression of metabolic pathways involved in carbohydrate metabolism [9,10], improving insulin sensitivity [3,11], or the translocation of GLUT-4 transporters [6,8,12]. In addition, phenolic compounds may act as antidiabetic molecules by improving lipid metabolism [13].

In this context, scientific interest in the potential benefits of *Hibiscus* plant extracts has noticeably increased in the last decades. *Hibiscus sabdariffa* is a plant belonging to the Malvaceae family. Whilst diverse parts of the plant can be used [2], the most used part is the calyx [14] because of its interesting chemical profile containing bioactive compounds such as phenolic compounds [15]. Among the phenolic compounds, diverse hydroxybenzoic and hydroxycinnamic phenolic acids (i.e., gallic acid, syringic acid, protocatechuic acid, caffeic acid, chlorogenic acid) and flavonoids such as epigallocatechin gallate, quercetin, myricetin, kaempferol, and diverse anthocyanins have been characterized in flowers [16]. *Hibiscus* can be consumed as infusions or be used as dietary ingredients [2] or as a pharmaceutical [17]. Some reports have indicated antioxidant and anti-inflammatory activities for *Hibiscus* extracts, as well as a potential antidiabetic capacity. Even though several in vitro studies have shown a potential inhibitory activity of the α-amylase enzyme [18,19], there is a lack of in vivo studies including animal models or clinical trials focused on this topic [16]. Moreover, the few studies already published observed different results depending on the used *Hibiscus* variety [2], indicating the need to investigate the potential effect of the different existing *Hibiscus* varieties. Thus, the aim of this research was to study the potential antidiabetic effects of extracts obtained from the creole variety of *Hibiscus sabdariffa* from the Morelia region (Mexico), using an induced diabetic lab rat model. This *Hibiscus* variety has been scarcely studied, and, to the best of our knowledge, its potential as a modulator of the glucose homeostasis in a diabetic lab rat model has not been investigated so far. This research pointed out that the studied *Hibiscus* variety from the Morelia region shows modulatory effects on the hyperglycemia condition, suggesting a potential use as a functional ingredient or biopharmaceutical compound.

## 2. Materials and Methods

### 2.1. Plant Material

Creole Jamaica flowers (*Hibiscus sabdariffa*) from the Morelia region (Mexico) were purchased from local organic crops in the Morelia region (Michoacán State, Mexico). Specifically, the flower samples were obtained from KIAN-ORGANC enterprise. The calyxes were manually separated and dried at 40 °C for four days. After that, the resulting dried calyxes were grounded in a commercial blender (Nutribullet NBR-0504S-DL, Personal Blender, Los Angeles, CA, USA) and sifted with a sieve of 200 μm. The obtained homogenized powder was stored in a sealed bottle, protected from light, and stored at −20 °C until further use.

### 2.2. Chemicals

Gallic acid, quercetin, 6-hydroxy-2,5,7,8-tetramethylchromane-2-carboxylic acid (Trolox), potassium persulfate, 2,2′-azinobis(3-ethylbenzothiazoline-6-sulphonic acid) diammonium salt (ABTS), 2,2-diphenyl-1-picrylhydrazyl (DPPH), Folin–Ciocalteu reagent, aluminum chloride, starch Assay Reagent (S9144), 3,5 Dinitro-2-hydroxybenzoic acid (DNS), α-amylase from porcine pancreas (A3176), streptozotocin (S0130) (STZ), ferrozine, hexamethylenetetramine, and ethylenediaminetetraacetic acid (EDTA) were purchased from Sigma-Aldrich (Saint Luis, MO, USA). All standards were of analytical HPLC grade and supplied by Sigma-Aldrich (Saint Louis, MO, USA) for quercetin, apigenin (purity ≥ 95%), myricetin (purity ≥ 96%), kaempferol, vanillic acid, protocatechuic acid (purity ≥ 97%), caffeic acid, p-coumaric acid (purity ≥ 98%), gallic acid, ferulic acid, and cinnamic acid (purity ≥ 99%). The acetic acid solution was of analytical grade for HPLC from Sigma-Aldrich (Saint Louis, MO, USA); hydrochloric acid 37% analytical reagent grade, sodium hydroxide, ethyl acetate, and diethyl ether were obtained from Meyer Chemical S.A de C.V (Ciudad de Mexico, Mexico); and acetonitrile, ethanol, and methanol were obtained from J.T. Baker Chemical Co (Phillipsburg, NJ, USA). Deionized water was used in all experiments.

Disodium carbonate, methanol, ethanol, acetone, citric acid, ethyl acetate, ferric chloride, sodium phosphate, sodium chloride, HCl (37%), and iron dichloride were obtained from Merk (Naucalpan de Juárez, Mexico).

### 2.3. Obtention of the Functional Ingredient from Hibiscus Leaves

The extract evaluated in the induced diabetic lab rat model was selected according to its chemical composition and functional properties. To achieve this, various solid–liquid extractions using hydroalcoholic solvents were conducted. The resulting extracts were analyzed in terms of chemical composition (total phenolic compounds, total flavonoids and total anthocyanins), as well as their functional properties, including antioxidant activity (conducted by ABTS and DPPH methods) and chelating capacity.

#### 2.3.1. Obtention of *Hibiscus* Extracts

To select the optimal extraction conditions, solid–liquid hydroalcoholic extractions using different solvent composition and extraction temperatures were conducted, while extraction time (24 h), pH (2.5), and particle size (5%, *w*/*v*) were maintained as constant factors. The extraction solvent pH was adjusted to pH 2.5 with citric acid. These extraction conditions were based on the report by Hapsari et al. [20]. For this purpose, 4 g of sample were mixed with 80 mL of the extraction solvent under the specific extraction condition, being solid–liquid extractions with water:ethanol 80:20, 50:50, or 100:0 (*v*/*v*) at 25 or 50 °C. After the extraction procedures, the obtained extracts were filtered using Watman paper No. 4. Subsequently, ethanol was removed by vacuum evaporation using an IKA RV 10 control evaporator (IKA, Staufen, Germany), and the samples were freeze-dried for 3 days using an Labcono FreeZone 2.5 L equipment (Labcono, Kansas, MO, USA). The resulting extracts were stored in sealed bottles, protected from light, and stored at −20 °C. It is important to indicate that hydroalcoholic extraction was selected as extraction solvents since hydroalcoholic solvents made with ethanol have been recognized as Generally Recognized as Safe (GRAS) solvents, thus ensuring the use of environmentally friendly extraction solvents [21].

#### 2.3.2. Total Phenolic Compounds

Total phenolic compounds (TPC) were evaluated according to Nieto et al. [22]. The Folin–Ciocalteau reagent method was used for the analysis, enabling the determination of TPC values in the *Hibiscus* extracts. Gallic acid served as the reference compound, and the results were expressed as mg of gallic acid equivalents (GAE)/g extract. Analyses were conducted in triplicate.

#### 2.3.3. Total Flavonoid Content

Total flavonoid content (TFC) of the extracts was determined according to Soares et al. [23], with some modifications. To do this, 400 mg of the extract were mixed with 1 mL of hexamethylenetetramine 0.5% (*w*/*v*), 20 mL of acetone, and 2 mL of hydrochloric acid. The mixture was stirred for 30 min and then filtered through a small piece of cotton wool. The resulting residues were washed twice with acetone (20 mL) while stirring for 10 min. Subsequently, the mixtures were filtered again and made up to 100 mL with acetone. Next, 20 mL of this solution were transferred to a decanting funnel, and three extraction cycles with 15 mL of ethyl acetate were performed. The organic phases (ethyl acetate) were mixed and washed twice with 50 mL of water. Then, the organic phase was made up to 50 mL with ethyl acetate, resulting in the stock solution (SS). Next, 10 mL of the SS solution was mixed with 2 mL of a 2% aluminum chloride solution in ethanol (AlCl3; *w*/*v*), and the solution was made up to 25 mL with a 0.5% acetic acid solution in methanol (*v*/*v*), obtaining the probe solution (PS). Simultaneously, 10 mL of SS was made up to 25 mL with the methanol/acetic acid solution, obtaining the contrast solution (CS). The absorbance of PS against CS was measured at 420 nm after 30 min. Samples were analyzed in triplicate, and the results were expressed as a percentage of TFC, using quercetin as a reference (with the specific absorption of the quercetin aluminum chloride complex considered as value of 500).

#### 2.3.4. Total Anthocyanin Content

Total anthocyanin content (TAC) was determined following the well-established method previously reported by Prior et al. [24]. The method consists of absorbance differences according to pH changes. To determine the total absorbance of the samples, measurements were conducted at 520 and 700 nm at pH 1.0 and 4.5., as follows:

Total Absorbance = (Abs_520_ − Abs_700_)_pH1.0_ − (Abs_520_ − Abs_700_)_pH4.5_ (1)

To estimate the TAC, cianidin-3-o-glucoside was used as the reference compound (molecular weight = 449.2 g/mol; molar extinction coefficient = 29,600). Results were expressed as milligram equivalents of cyanidin-3-o-glucoside (mg/g extract).

#### 2.3.5. Antioxidant Activity

Two different methods for assessing the antioxidant activity i.e., 2,2′-azino-bis (3-ethylbenzothiazoline-6-sulfonic acid (ABTS) and 2,2-diphenyl-1-picryl-hydrazyl-hydrate (DPPH) were employed.

The ABTS method was applied following the well-established method of Re et al. [25]. On the other hand, the DPPH method was applied following the well-established method of Brand-Williams et al. [26]. Trolox was used as the reference compound, allowing expressing the results as TEAC value, that is, as mmol Trolox equivalent/g extract. All analyses were conducted in triplicate.

#### 2.3.6. Metal Ions Chelating Activity

The chelating capacity of the extracts was determined based on the method reported by Xie et al. [27], with few modifications. This method analyzes the formation of a ferrozine complex, characterized by a purple color with a maximum absorbance at 562 nm. The chelating capacity of the extracts reduces the complex formation, decreasing the absorbance at 562 nm. Briefly, 1 mL of the sample solution was mixed with 0.05 mL of iron dichloride solution (2 mM) and 1.85 mL of ultrapure water. Then, 0.1 mL of ferrozine solution (5 mM) was added, and the mixture was vigorously stirred and maintained at room temperature for 15 min. Afterwards, the absorbance was determined at 562 nm. A control sample was conducted, using ultrapure water instead of extract sample solutions. Samples were analyzed in triplicate, and the chelating effect was calculated as follows:

Chelating effect (%) = (Abs_control_ − Abs_sample_)/(Abs_control_) × 100 (2)

### 2.4. In Vitro Inhibitory Activity of the α-Amylase Enzyme

The inhibitory capacity of the selected extract against α-amylase activity was determined according to the method reported by Ademiluyi and Oboh [28], with few modifications. A sodium phosphate solution (0.02 M) was prepared and adjusted to pH 6.9 with sodium chloride (0.006 M). An enzymatic solution of porcine pancreatic α-amylase (EC 3.2.1.1; 0.5 mg/mL) was prepared using the aforementioned phosphate solution. Additionally, a solution of starch at 0.5% was prepared in the phosphate solution. Then, 500 µL of the fresh enzymatic solution was heated at 37 °C for 10 min. Furthermore, a control sample without extract was prepared. Afterward, 500 µL of the starch solution was added, and the reaction was maintained for 20 min at 37 °C. Finally, the reactions were stopped by adding 1 mL of a dinitrosalicylic solution (1% *w*/*v*). The resulting mixture was made up to 10 mL in ultrapure water, and the absorbance was measured at 540 nm. Samples’ analyses were conducted in triplicate, and the inhibitory capacity was expressed as % of enzyme activity inhibition, as follows:

% Inhibition = (Abs_control_ − Abs_sample_)/(Abs_control_) × 100 (3)

### 2.5. Identification and Quantification of Phenolic Compounds by RP-HPLC

HPLC with a diode array detector (Waters 2996, Milford, MA, USA) and autosampler (Waters 717, Milford, MA, USA) was used. The analysis was carried out on a Symmetry C18 column (5 µm × 4.6 mm × 250 mm, WAT054275, Waters, Wexford, Ireland). The mobile phase (A) consisted of acetonitrile (JT Baker, Phillipsburg, NJ, USA), and the mobile phase (B) consisted of deionized water at pH 2.8 adjusted with HPLC grade acetic acid (Sigma-Aldrich, Saint Louis, MO, USA), with a flow rate of 1 mL/min and an injection volume of 20 µL. For the elution of flavonoids, the gradient conditions were as follows: 0 min 100% B; 0–10 min 35% B; 10–15 min 20% B; 15–16 min 5% B; 16–17 min 4% B; 17–18 min 80% B; 18–19 min 100% B; and 19–20 min 100%. UV-vis spectra were used to detect compounds using reference standards (Sigma Aldrich, Saint Louis, MO, USA). The absorbance for myricetin, quercetin, apigenin, and kaempferol were 252, 254, 266, and 265 nm, respectively. For phenolic acids, the gradient used was the following: 0 min 100% B; 0–15 min 50% B; 15–16 min 50% B; 16–18 min 35% B; 18–20 min 5% B; 20–21 min 4% B; 21–22 min 80% B; 22–23 min 100%; and 23–24 min 100% B. Post-run time was 6 min. The absorbance for gallic, caffeic, vanillic, coumaric, ferulic, protocatechuic, and cinnamic acids were 269, 323, 260, 309, 322, 259, and 274 nm, respectively. Three injections were made for each sample; the quantification was based on the relation to the area found compared to the standard using calibration curves and retention times. The limit of quantification (LOQ) was 15.6 µg/mL for flavonoids and 8.9 µg/ mL for phenolic acids. Chromatograms were recorded and analyzed using Empower Personal software version 6.01.2154.026.

### 2.6. Evaluation of Functionality in Induced Diabetic Rat Lab Model

**Figure 1:**
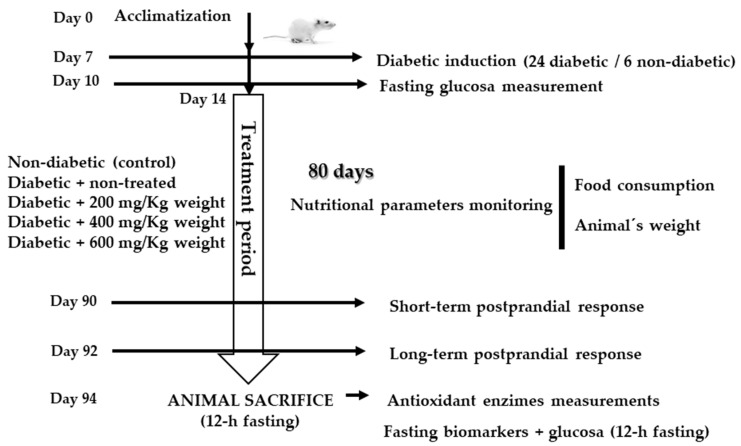
Whole experimental design for the extract effect evaluation with the animal model.

Once the optimal extraction conditions were determined (water:ethanol 80:20 (*v*/*v*) at 50 °C), the antidiabetic functional properties of the selected extract were evaluated in a lab rat model with induced diabetes. The outline of the experimental design is shown in Figure 1.

#### 2.6.1. Ethical Clearance

All animal experiments, procedures, and techniques used in this study adhered to the Official Mexican Normative NOM-062-ZOO-1999. The investigation plan (protocol code CIECUAL/010/2019) was submitted to and approved by the Ethics Committee of the Bioterio del Instituto de Ciencias de la Salud of the Universidad Autónoma del Estado de Hidalgo (CIECUAL). The study also followed the guidelines for the care and management of laboratory experimental animals of the National Academy of Science of the United States.

#### 2.6.2. Animal Acquisition and Husbandry

Healthy Wistar male rats aged 10–12 weeks were used for this experiment. The rats were kept in the Bioterio del Instituto de Ciencias de la Salud of the Universidad Autónoma del Estado de Hidalgo (Bioterio of the Health Science Faculty of the Autonomous University of Estado de Hidalgo). The rats were placed in 6 mm thick polycarbonate cages (32 cm × 47 cm × 20 cm). Wood shavings were used as bedding. Husbandry conditions consisted of normal ambient laboratory conditions, that is, temperature of 22 ± 2 °C, humidity, and a 12 h dark–light cycle.

Animal feeding and water access were performed *ad libitum*. All the animals were fed with a standard rodent pelleted diet chow 5001 (LabDiet, Sant Louis, MO, USA), allowing an isocaloric diet comprised of proteins (23.0%), lipids (4.5%), fiber (6.0%), minerals (8.0%), and carbohydrates (49%). Consequently, total calorie intake was provided mainly by carbohydrates (58%), whereas lower contributions were due to proteins (28.5%) and lipids (13.5%). The extract was administered once daily (in the morning) to each corresponding experimental group via oral gavage using a cannula.

#### 2.6.3. Establishment of Streptozotocin (STZ) Diabetes in Rats

Rats were given one week of acclimatization. After that, experimental type 1 diabetes mellitus was induced in healthy male rats (200–220 g), which were maintained with fasting conditions overnight. Administration of streptozotocin (STZ) at 60 mg/kg of the body weight was conducted by intraperitoneal puncture [29]. To this purpose, STZ was dissolved in a citrate buffer (0.1 M, pH 4.5) at 40 mg/mL (specific volume for each animal as applied to providing 60 mg/kg of the body weight, ranging between 0.32 and 0.40 mL). Also, a non-diabetic rat group (ND) was administered an intraperitoneal of the same mentioned citrate buffer (specific volumes were applied to each animal based on their weight). One week after the diabetes induction, all the experimental animals were maintained under fasting conditions for 72 h, and blood samples were collected to measure the plasma glucose levels, and therefore, to determine the hyperglycemic grade. Animals with plasma glucose levels exceeding 14 mmol/L were considered diabetic models. Finally, induced diabetic rats were randomly divided into four groups composed of 6 rats (n = 6). This sample size was chosen according to the criteria reported by Zhou et al. [29] and Kim et al. [30]. For that reason, the following experimental groups were created: non-diabetic (ND), non-treated diabetic or control diabetic (DC), and the three treated diabetic groups, that is D200 (treated with 200 mg/kg rat’s weight), D400 (treated with 400 mg/kg rat’s weight), and D600 (treated with 600 mg/kg rat’s weight). The experimental conditions were maintained for 80 days in all the experimental groups. Plasma glucose levels (under fasting conditions) were measured at the initial and end experimental points, while glycosylated hemoglobin, cholesterol, triglycerides, high-density lipoproteins (HDL), very low-density lipoproteins (VLDL), and low-density lipoproteins (LDL) were analyzed at the end of the experimental period (as explained below).

#### 2.6.4. Plasma Collection

After 80 days under the specific experimental conditions, all rats were subjected to 12 h fasting conditions. Subsequently, the animals were anesthetized with sodium pentobarbital (1 g/L) and blood samples were collected (through cardiac puncture) into tubes containing anticoagulants to avoid sample hemolysis. Blood plasma was obtained by centrifuging the blood samples for 10 min at 10,000 rpm, being immediately stored at −80 °C until further analysis.

#### 2.6.5. Nutritional Parameters

To analyze the potential impact of the extracts on the nutritional parameters, considering the physiological condition (diabetic or non-diabetic), three commonly measured nutritional parameters were monitored throughout the experimental period for all experimental groups: body mass gain (BMG), specific growth rate (SGR), and metabolic growth rate (MGR), as described by Kumar et al. [31]. These nutritional parameters were calculated according to the equations given below:

BMG % = [Final body mass (FBM) − initial body mass (IBM)/IBM] × 100(4)

SGR (% per days) = [(ln FBM in g) − (ln IBM in g)/number trial days] × 100(5)

MGR (g kg^0.8^ /day) = (BMG g)/{[(IBM g/1000)^0.8^ + (FBM g/1000)^0.8^]/2}/duration of the trial days (6)

#### 2.6.6. Biochemical Parameters

The collected plasma samples of all groups of animals at the end of the experimental period after the fasting condition (12 h) were analyzed in terms of glucose, glycosylated hemoglobin, cholesterol, triglycerides, HDL, VLDL, and LDL. A commercial kit was used to quantify these parameters by enzymatic–colorimetric methods, according to the manufacturer’s instructions (Wiener Laboratories S.A.I.C., Buenos Aires, Argentina), excepting the glycosylated hemoglobin, which was measured using the kit Stanbio Glicohemoglobina (Pre-Fil) (Stanbio Laboratory, Boerne, TX, USA).

### 2.7. Glucose Homeostasis Analyses

#### 2.7.1. Short-Term Postprandial Glucose Response

After the whole experimental period (12 weeks), the animals were subjected to a 12 h fasting period prior to the assessment of the short-term postprandial response, as previously reported by our group [32]. After that, animals from all the experimental groups were allowed to feed *ad libitum* for 30 min. Afterward, food was removed. Blood samples (0.5 mL) were collected every 30 min for 150 min via capillary puncture of the tail saphenous vein. Collected samples were placed in tubes containing heparin as an anticoagulant to avoid hemolysis of the sample. Subsequently, they were centrifuged for 5 min at 5000 rpm to obtain the plasma, which was stored freezing at −80 °C until further analysis. Glucose concentrations were evaluated using a commercial kit by enzymatic–colorimetric kit following the manufacturer’s protocol (Wiener Laboratories S.A.I.C. brand).

#### 2.7.2. Long-Term Postprandial Glucose Response

To evaluate the effect of the *Hibiscus* extracts in the long-term postprandial glycaemia, the same procedure described above was performed, with sample collection conducted every hour for 7 h. Plasma glucose levels were quantified using the same commercial enzymatic–colorimetric kit (Wiener Laboratories S.A.I.C.).

#### 2.7.3. Insulin Resistance Index

Potential insulin resistance was estimated according to the triglyceride–glucose (TyG) index [33]. This index mathematically estimates the potential insulin insensibility as the product of fasting plasma concentrations of triglycerides and glucose, expressed on a logarithmic scale (values above 8.7–8.8 are considered indicative of insulin resistance), calculated as follows:

TyG = Ln [fasting triglycerides (mg dL) × fasting glucose (mg dL)/2](7)

### 2.8. Statistical Analyses

Statistical analyses of the results were conducted using the software Statgraphics Centurion XVI (Statistical Graphics Corp., Warrenton, VA, USA). One-way ANOVA, along with Tukey tests (significance level of *p* ≤ 0.05), was used for mean comparisons.

## 3. Results

### 3.1. Obtention of the Functional Ingredient of Hibiscus Extracts

#### 3.1.1. Chemical Characterization of the *Hibiscus* Extracts

The studied extraction conditions resulted in extracts with different composition profiles. The results of the chemical composition of the extracts in terms of TPC, TFC, TAC, TBL, and TBX are shown in Table 1. The extractions conducted with water:ethanol 80:20 (*v*/*v*) allowed the extracts with the highest TPC values, regardless the applied temperature (no significant statistical differences were found between both extracts), being noticeably higher compared with the other obtained extracts. On the opposite side, the extractions conducted with water:ethanol 50:50 (*v*/*v*) resulted in the lower TPC values. Conversely, the highest TFC was obtained with water at 50 °C, whereas extractions conducted with water:ethanol 80:20 (*v*/*v*) at 25 °C allowed superior TAC values.

#### 3.1.2. Antioxidant Activity of the *Hibiscus* Extracts

The antioxidant capacity of the diverse extracts obtained using the ABTS and DPPH methods is shown in Table 1. The antioxidant activity measured with both methods showed, in general, the same trend as the TPC values. Then, higher antioxidant activities were observed in the extracts conducted with water:ethanol 80:20 (*v*/*v*) regardless the applied temperatures.

#### 3.1.3. Chelating Capacity of the *Hibiscus* Extracts

The chelating capacity of the extracts showed significant differences (Table 1). Conversely to the antioxidant capacity trend, water:ethanol 80:20 (*v*/*v*) resulted in the lowest chelating capacity. However, the water extraction at 50 °C exhibited the maximum cheating capacity, although no significant differences were determined compared to the extracts obtained at 25 °C, nor with the water:ethanol 50:50 (*v*/*v*) extracts regardless of the extraction temperature.

#### 3.1.4. Identification and Quantification of Phenolic Compounds by RP-HPLC

The optimal extract (water:ethanol 80:20 (*v*/*v*) at 50 °C) was analyzed by HPLC-RP to determine the phenolic composition of the sample (Table 2). Diverse phenolic acid and flavonoids were identified in the extract. Among them, flavanols were the most abundant, mainly as myricetin. However, quercetin and kaempferol aglycones were not found in the sample (Figure S1).

On the other hand, various phenolic acids were identified. Gallic acid was the most abundant phenolic acid, together with caffeic and ferulic acids. Conversely, neither vanillic acid nor protocatechuic acid were observed in the sample, whereas cinnamic acid was determined under the quantification limit of the method. Additionally, diverse compounds with absorbances at 520 nm were observed in the sample, confirming the presence of diverse anthocyanins, as was previously quantified in the sample (Table 1).

### 3.2. Inhibitory α-Amylase Activity of the Selected Hibiscus Extract

**Figure 2:**
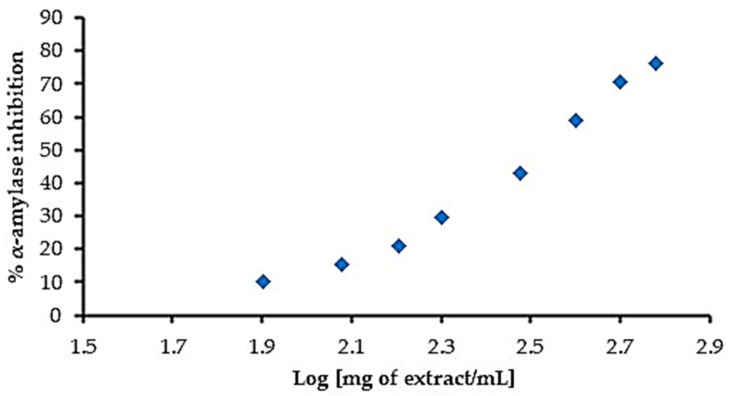
Inhibitory capacity against the enzyme α-amylase activity of the obtained *Hibiscus* extracts (obtained with water:ethanol 80:20 *v*/*v*, at 50 °C).

Once the chemical composition was analyzed, the extraction conditions selected were water:ethanol 80:20 (*v*/*v*) at 50 °C, as it showed the highest TPC values, as well as the highest antioxidant activity by ABTS and DPPH. Consequently, extractions under these conditions were conducted and the inhibitory capacity of the extracts against the α-amylase enzyme was evaluated. The extract showed a linear dose-dependent activity, with higher inhibitory activity at higher extract concentrations. Results are shown in Figure 2. The IC50 value, that is, the extract concentration that inhibits 50% of the enzyme activity, was determined as 323.66 μg/mL.

### 3.3. Effect of the Hibiscus Extracts on Nutritional Parameters

Before the nutritional parameters analyses (BMG, SGR, and MGR), the amount of food along the experimental period was assessed. In general, similar amounts of food were consumed by the diverse experimental groups, with slightly higher amounts for the non-diabetic group (ND). However, when considering the mean amount of consumed food per kg of weight (mean consumed food in g/kg of rat weight per day), the ND group showed consumption significantly lower than the diabetic groups, regardless of the level of *Hibiscus* extract intake (Table 3). Then, in all the diabetic groups a lower growth rate was observed compared to the ND group.

The weight gain and body development of the animals during the experimental period were monitored to analyze the effect of ingredient consumption on nutritional parameters. The results of nutritional parameters are shown in Table 3. The ND showed weight gain and body development in accordance with the animal physiology. Conversely, the diabetic groups showed lower growth and body development values. In this regard, the animals of the diabetic control diabetic group (DC) showed the lower development trend in terms of BMG, SGR, and MGR parameters, although no differences were found between the DC group and the diabetic animals fed with 200 and 400 mg/kg weight (D200 and D400 groups, respectively). Moreover, no significant differences in BMG and SGR values between the ND group and the diabetic group fed with 600 mg/kg weight were observed. D600, D400, and D200 groups exhibited higher weight increases compared to the DC group. Therefore, the consumption of 600 mg/kg showed improvements in the nutritional parameters of BMG and SGR.

### 3.4. Effect of the Hibiscus Extracts on Metabolic Biomarkers

To elucidate the effect of the *Hibiscus* extract consumption on glycaemia levels, the fasting plasma glucose levels were determined before and after the experimental period. Thus, 72 h after diabetic induction in the animals by STZ injection, plasma glucose levels were measured to ensure that the pathological condition was induced. The groups of diabetic animals showed a plasma glucose level higher than 14 mmol/L after 72 h, with similar baseline glucose concentrations (26.41 ± 3.33, 34.33 ± 2.66, 32.29 ± 2.24 and, 31.92 ± 2.08 mmol/L for DC, D200, D400 and, D600, respectively), although the levels of the DC were slightly lower. However, the ND group resulted in normal glycaemia (4.81 ± 0.62 mmol glucose/L). These results confirm the induction of a diabetic condition within DC, D200, D400, and D600 groups. At the end of the study (12 weeks), the glucose concentrations after 12 h of fasting condition in the ND group (5.47 ± 0.57 mmol glucose/L) were lower compared to the diabetic groups. Nevertheless, the glucose plasma levels of the diabetic groups remained steady over normal physiological values regardless of the applied treatment (DC, D200, D400, and D600 groups) (Table 4). It is important to point out that no significant improvements in plasma glucose levels were observed in the DC, whereas for the diabetic treated groups plasma glucose levels were reduced in a dose-dependent manner, with higher reductions in glucose level observed in D600 group. Then, the treated groups exhibited a significant reduction in plasma glucose levels compared to the initial levels of 35.2%, 41.63%, and 50.1% for D200, D400, and D600 group, respectively (*p* ˂ 0.05).

On the other hand, related to the glycaemia management, at the end of the study the % of glycosylated hemoglobin in all experimental groups was measured (Table 4). No significant differences in the hemoglobin glycosylation levels between the ND group, 7.5% ± 0.87% and the treated groups D200, D400, and D600 (8.1% ± 0.87%, 7.5% ± 1.12%, and 7.4% ± 1.0%, respectively) were found, whereas the diabetic DC group showed noticeable higher values (14.9% ± 1.76%).

Lipid (triglycerides) and cholesterol plasma levels are biomarkers widely associated with metabolic syndrome. In this study, significant differences in total triglycerides, cholesterol, VLDL, LDL, and HDL plasma levels were observed, and consequently, in the LDL/HDL index. Results are shown in Table 5. In general, total triglycerides, HDL, and VLDL were similar between the ND group and the D600 treated group, whereas total cholesterol and LDL levels were reduced in the D600 treated group. As a result, a reduced LDL/HDL index was observed in the three treated groups compared to ND group, but also compared with the DC group. Hence, the treated group, and especially the D600 group, showed a decreasing trend in lipid triglycerides and cholesterol, compared to the DC group. These results suggest a potential modulatory capacity of the *Hibiscus* extracts in lipid metabolism.

### 3.5. Effect of the Hibiscus Extracts on Glucose Homeostasis: Postprandial Response

**Figure 3:**
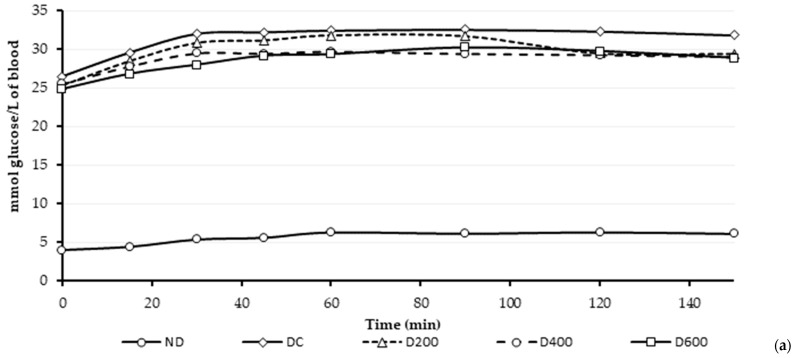

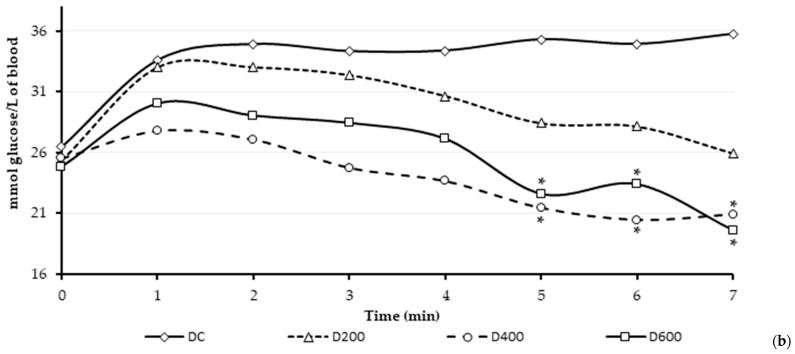
Postprandial response of the diverse experimental groups after the whole experimental period (80 days). (**a**) Short-term postprandial response; (**b**) Long-term postprandial response. * Indicate significant differences between treatment and diabetic control group.

After the whole experimental period (12 weeks), the animals were maintained in a 12 h fasting state. Afterwards, animals were allowed *ad libitum* feeding, after which fasting was re-introduced. The postprandial glucose response was monitored in the short term (for 180 min) and in the long term (for 7 h). The results are represented in Figure 3. To enhance the comparison of the postprandial glucose response, the areas under the curve (AUC) were calculated and compared. Because of ethical reasons, long term postprandial glucose was not monitored in the ND group since a normal glycemic response was previously confirmed during the short-term postprandial response for this group, thus minimizing animal suffering.

The short-term postprandial kinetics showed a typical animal physiological response in the non-diabetic animals (a small plasma glucose peak close to 30 min and returning to normal glycaemia). Conversely, the diabetic groups showed a typical diabetic response, despite the applied treatment with the extract. Nevertheless, reduced values of plasma glucose levels were determined at 150 min in the treated groups (29.41 ± 2.74, 29.01 ± 4.56, and 28.88 ± 3.70 mmol/L, to D200, D400, and D600, respectively) compared to DC group (31.83 ± 4.55 mmol/L). Then, at the end point of the experiment, the treated groups reduced the hyperglycemia condition between 7 and 9% compared to the DC group. However, comparison of the AUC revealed no significant differences among the treated groups, neither between them nor the DC group.

Regarding the long-term postprandial kinetic response, noticeable differences between the diabetic treated groups and the diabetic non-treated group were found. On that account, at the end point of the experiment, the D200 group reduced the glucose levels 27.4% compared to the DC group, whereas similar decreases for D400 (41.5%) and D600 (45.1%) groups were observed. However, no significant differences were determined in the AUC of D200 and DC groups; meanwhile, D400 and D600 resulted in significantly lower AUC compared to the DC group.

### 3.6. Effect of the Hibiscus Extracts on Glucose Homeostasis: Insulin Resistance Index

Values above 8.7–8.8 in the triglycerides and glucose index (TyG index) have been suggested as a biomarker of insulin resistance, indicating pre-diabetic and diabetic conditions [33]. In this study, the ND group showed an insulin index of 8.43 ± 0.19 after the experimental period, whereas values above 8.8 were determined for all the diabetic groups (Table 3). However, a significant, scarce improved prognostic for the treated groups (10.49 ± 0.35, 10.22 ± 0.27, and 10.17 ± 0.34, for D200, D400, and D600, respectively) compared to the DNT group (11.02 ± 0.14) was observed. These results are in concordance with the improved glucose plasma levels under fasting conditions. Accordingly, the results suggest that consumption of the *Hibiscus* extracts may improve insulin resistance under diabetic conditions, although healthy levels cannot be achieved.

## 4. Discussion

In the present study, hydroalcoholic extracts of the calyxes of the creole variety of *Hibiscus sabdariffa* from the Morelia region (Mexico) were evaluated as a bioactive compound source and, for the first time, as an antidiabetic extract in a lab rat model with an induced diabetic condition.

The hydroalcoholic extracts obtained from *Hibiscus sabdariffa* (Morelia) exhibited higher antioxidant capacities compared to the aqueous extracts. It has been postulated that hydroalcoholic solvents enhance the recovery of phenolic compounds, leading to higher TPC and antioxidant extracts [21]. Previous studies have demonstrated that hydroalcoholic extractions are adequate solvents to obtain antioxidant or functional extracts from *Hibiscus* sources [34,35]. The obtained extracts showed TPC values ranged between 189.15–405.06 mg GAE/g extract, and antioxidant capacity ranged between 0.043–0.219 mmol Trolox/g extract by ABTS and 0.031–0.13 mmol Trolox/g extract by DPPH. Considering that in this study extraction yields ranged from 37.4 to 57.53%; these results are in concordance with previous research. Mercado-Mercado et al. [36] found a TPC value of 63.65 mg GAE/g dry calyx of *Hibiscus sabdariffa*. Magaña-Rodríguez et al. [5] reported a TEAC value of *Hibiscus rosa sinensis* extracts of 0.293 mmol trolox/mL extract (DPPH) and 0.443 mmol trolox/mL extract (ABTS). In this context, the extracts obtained with water:ethanol (80:20, *v*/*v*) at 50 °C (but also at 25 °C) resulted in the highest TPC values, as well as the highest antioxidant capacity regardless of the antioxidant method used. These results indicate that the antioxidant activity was due to the TPC.

Regarding the phenolic composition of the optimal extract, diverse phenolic acids were identified in the sample. The samples resulted in a lack of protocatechuic acid, although this compound has been little reported in some *Hibiscus* samples [37]. Gallic, ferulic and caffeic acid were also previously determined in *Hibiscus* extracts, where gallic and caffeic acid resulted as the main phenolic acids, being in concordance with our results [38]. On the other hand, although myricetin and quercetin have been identified among the most abundant flavanols in *Hibiscus* extracts [37], in our research we found myricetin as the main flavanol, whereas quercetin aglycone was not detected. Besides, apigenin was observed in the sample. Apigenin has been little reported as a phenolic compound in *Hibiscus* samples [38], being in concordance with our results, although noticeable amounts were observed in this study. In the sample were also measured anthocyanin compounds (total anthocyanins). In this regard, it is well known that *Hibiscus* extracts show great amounts of anthocyanins, mainly cyanidin-3-sambubioside and delphinidin-3-sambubioside but also delphinidin-3-glucoside and cyanidin-3-glucoside [39].

In the present study, the intake of the *Hibiscus* extract under diabetic conditions showed clear beneficial effects in the lab rats. Regarding the nutritional parameters, the intake of the *Hibiscus* extract was not able to completely ameliorate the diabetic condition, affecting the animal’s development, and therefore, showed the animal’s reduced growth as a consequence of the diabetic condition. Sachdewa et al. [40] observed an improved development of induced animals treated with *Hibiscus rosa sinensis* extracts compared with the diabetic control group (untreated), agreeing with the highest doses treatment of the present study (600 mg/Kg). Conversely, Adeyemi and Adewole, [41] did not observe differences in body weight and growth between treated diabetic rats with *Hibiscus sabdariffa* extracts and healthy rats. However, it is important to consider that noticeably higher doses were given to the animals in the mentioned study. Also, this result is aligned with the observed trend in the present investigation, where the rats treated with 600 mg/kg showed no significant differences in BMG and SGR parameters with the ND group, approaching a healthier condition. It has been previously reported that *Hibiscus* extracts are able to improve the diabetic condition through diverse potential mechanisms, mainly derived from the antioxidant activity and their bioactive compounds. In this context, the flavonoid fraction of *Hibiscus sabdariffa* extracts can induce β-cell regeneration, as well as improve the diabetic condition due to their antioxidant activity [41]. Sachdewa et al. [40] suggested a potential reduction of the gluconeogenic enzymes (glucose-6-phosphatase), as well as an improvement of insulin release, with *Hibiscus rosa sinensis* extract treatment in diabetic rats. Also, hydroalcoholic extracts from *Hibiscus rosa sinensis* can regenerate the damaged pancreatic β cells [42]. The results of this study suggest that the improvement of glucose management derived from the extract intake is not able to completely ameliorate the diabetic condition but can enhance energy management, probably derived from an improved glucose metabolism or management. These results agree with the inhibitory capacity of the α-amylase activity observed for the extracts, as has been previously reported for other *Hibiscus* extracts [5,19]. In this regard, the inhibitory capacity was noticeably superior that the capacity showed by extracts obtained from cultivars Aswan and Sudan-1 [18]. The inhibitory capacity has been associated with the activity of the phenolic compounds [2], such as gallic or protocatechuic acids, with gallic acid being the main phenolic acid of the extract [19]. It is important to point out that the *Hibiscus sabdariffa* extracts (Morelia) of the present study showed slight improvements in the short-term postprandial condition, whereas significant improvements were observed regarding long-term postprandial responses, although only the highest doses (D400 and D600 groups) were able to significantly improve the diabetic condition. Similar results were reported for hydroalcoholic extracts obtained from *Hibiscus rosa sinensis*, showing a long-term postprandial response improvement in a dose-dependent manner; therefore, higher extract doses resulted in lower glucose serum levels [42]. Consequently, regardless of the intake amount of the extract, all the treated groups of the present study showed reduced glucose plasma levels under fasting conditions, agreeing with the long-term postprandial results. These results were especially noticeable at the highest tested dose (600 mg/kg), allowing the reversal of the glucose levels close to 50% compared to the DC group. Other investigations have also observed improved glucose serum levels under fasting conditions, being in concordance with the present study. The consumption of 600 mg/kg of root extracts from *Hibiscus rosa sinensis* allowed the reversion of glucose levels in the diabetic condition by 30% [31]. Also, because of the improved glucose management with the extract intake, lower glycosylated hemoglobin levels were determined. The improvement of glucose management can be associated not only with decreased α-amylase activity (as mentioned before) but also with reduced glucose intake during the digestion process, probably modulated by a competitive mechanism with glycosylated anthocyanins [8]. Additionally, phenolic compounds such as anthocyanins can exert the oxidative stress on islet cells against hyperglycemia through the AMPK/ACC/mTOR pathway, as well as cyanidin-3-glucoside protected cells from high glucose-induced oxidative stress by activating glutathione synthesis [11].

In this study, an evident improvement in lipid metabolism in the animals treated with the *Hibiscus* extract at doses of 600 mg/kg was observed. These results suggest a potential impact of the *Hibiscus* extract compounds (such as phenolic compounds) in the lipid management of the liver. Kumar et al. [31] observed that induced diabetic rats reduced the total cholesterol and total triglycerides when animals were treated with *Hibiscus rosa sinensis* extracts, enhancing the lipid metabolism, agreeing with this study. Sachdewa et al. [40] observed enhanced lipid metabolism in diabetic rats fed with *Hibiscus rosa sinensis* extracts, characterized by reductions in cholesterol (close to 22%) and triglycerides (close to 30.5%), while increasing HDL levels (close to 12.5%) compared with diabetic non-treated rats. Comparable results were observed in the present study at high doses. In this context, previous investigations have pointed out that *Hibiscus sabdariffa* extracts are able to reduce the lipid peroxidation in the liver of diabetic individuals due to its antioxidant activity, as well as ameliorate the liver damage [43]. The improvement of the lipid management derived of the intake of *Hibiscus* extracts or infusions, mainly observed as reductions in LDL but also increases in HDL levels, has been associated with their phenolic composition, such as diverse anthocyanins, among them delphinidin- 3-sambubioside and cyanidin-3-sambubioside [44]. As a direct consequence of the glucose and lipid improvements, the intake of the extracts allowed slight improvements in TyG index (insulin resistant) in the present study, although it did not allow healthy values to be achieved.

## 5. Conclusions

The results of this study suggest that a dose of 600 mg/kg of the hydroalcoholic extracts (water:ethanol 80:20 (*v*/*v*) at 50 °C) from the *Hibiscus sabdariffa* creole variety from Morelia (Mexico) can impact lipid and glucose metabolism, showing improvements in long-term glucose postprandial management and fasting glucose glycaemia, as well as promoting the reduction in total cholesterol, triglycerides, VLDL, and LDL. These results are aligned with the general trend of the antidiabetic properties observed for hibiscus (*Hibiscus sabdariffa* or *Hibiscus rosa sinensis)* extracts, although few experiments have been conducted in animal models or clinical trials but mainly reported as inhibitory capacities against α-amylase activity. Since more evidence is necessary in animal models, and mainly in human clinical trials, the present study contributes to increase the evidence in in vivo research of antidiabetic capacities derived from *Hibiscus* extract intake. However, further studies focused on the impact of the consumption of the obtained extracts on glucose homeostasis (hepatic gluconeogenesis regulations and muscle glucose intake) are necessary. Additionally, studies focused on elucidating the exact mechanism of action at the molecular level of the specific bioactive compounds of the extracts are necessary. Nevertheless, this study highlights *Hibiscus sabdariffa* from Morelia as a potential source for the obtention of extracts with antidiabetic and antioxidant capacities. Nevertheless, these extracts could be used as ingredients for nutraceutical or functional food formulations, benefiting both diabetic individuals and the healthy population.

## Figures and Tables

**Table 1:** Chemical characterization (TPC = total phenolic content; TFC = total flavonoid content; TAC = total anthocyanin content), antioxidant activity (T. = trolox), and chelating capacity of the *Hibiscus* extracts.

Extraction Condition	TPC (mg GAE/g)	TFC (mg QE/g)	TAC (mg CE/g)	ABTS (μmol T./g)	DPPH (μmol T./g)	Chelating (%)
Water:ethanol 80:20 25 °C	405.06 ± 7.07 ^a^	23.98 ± 0.75 ^b^	9.00 ± 0.01 ^a^	219.4 ± 0.03 ^a^	134.4 ± 0.01 ^a^	40.93 ± 0.03 ^b^
Water:ethanol 80:20 50 °C	403.28 ± 7.71 ^a^	25.30 ± 1.58 ^b^	4.80 ± 0.05 ^c^	192.5 ± 0.01 ^a^	132.0 ± 0.01 ^a^	41.64 ± 0.02 ^b^
Water:ethanol 50:50 25 °C	202.50 ± 2.04 ^d^	15.96 ± 0.24 ^d^	5.40 ± 0.08 ^b^	77.9 ± 0.01 ^c^	42.7 ± 0.01 ^b^	49.99 ± 0.03 ^a^
Water:ethanol 50:50 50 °C	189.15 ± 5.04 ^e^	17.82 ± 0.86 ^d^	3.00 ± 0.01 ^d^	170.5 ± 0.01 ^ab^	44.6 ± 0.01 ^b^	51.97 ± 0.01 ^a^
Water:ethanol 100:0 25 °C	232.78 ± 2.31 ^c^	20.38 ± 0.82 ^c^	3.60 ± 0.04 ^d^	110.9 ± 0.01 ^b^	31.2 ± 0.01 ^b^	50.73 ± 0.03 ^a^
Water:ethanol 100:0 50 °C	312.46 ± 9.84 ^b^	28.86 ± 1.54 ^a^	4.80 ± 0.02 ^bc^	42.7 ± 0.01 ^c^	54.1 ± 0.01 ^b^	53.37 ± 0.04 ^a^

^a,b,c,d,e^ Different letters indicate significant differences between samples by Tukey post-hoc test (*p* ≤ 0.05), expressed as mean value ± S.D.

**Table 2:** Phenolic composition of the extract identified and quantified by HPLC-RP (mean values ± S.D.).

Compound	mg/g 100 Extract
Gallic acid	5.29 ± 0.24
Caffeic acid	4.15 ± 2.11
Vanillic acid	N.D.
p-Coumaric acid	2.15 ± 0.27
Ferulic acid	4.22 ± 0.41
Protocatechuic acid	N.D.
Cinnamic acid	<LOQ
Myricetin	18.79 ± 3.26
Quercetin	N.D.
Apigenin	10.16 ± 1.93
Kaempferol	N.D.

N.D. = No Detected. <LOQ = under quantification limit.

**Table 3:** Average food consumption (g food/kg weight · day) of the experimental groups along the experimental period, and nutritional parameters of the different experimental groups.

Experimental Group	BMG (%)	SGR (%)	MGR (g/kg · día)	Daily Intake (g Food/kg · día)
ND	33.05 ± 3.87 ^a^	0.36 ± 0.04 ^a^	2.95 ± 0.28 ^a^	75.94 ± 8.39 ^b^
DC	10.04 ± 9.34 ^b^	0.12 ± 0.11 ^b^	0.87 ± 0.82 ^b^	116.83 ± 14.48 ^a^
D200	13.14 ± 10.64 ^b^	0.15 ± 0.12 ^b^	1.15 ± 0.91 ^b^	128.79 ± 10.22 ^a^
D400	16.48 ± 6.80 ^b^	0.19 ± 0.07 ^b^	1.46 ± 0.58 ^b^	107.80 ± 7.11 ^a^
D600	19.01 ± 10.20 ^ab^	0.21 ± 0.10 ^ab^	1.65 ± 0.80 ^b^	116.29 ± 13.36 ^a^

^a,b^ Different letters indicate significant differences between samples by Tukey post-hoc test (*p* ≤ 0.05), expressed as mean value ± S.D. BMG = body mass gain; SGR = specific growth rate; MGR = metabolic growth rate. ND = non-diabetic group; DC = control diabetic group; D200 = diabetic group treated with 200 mg/kg; D400 = diabetic group treated with 400 mg/kg; D600 = diabetic group treated with 600 mg/kg.

**Table 4:** Baseline (before the experimental period, 0 days) and final (after the experimental period, 80 days) plasma glucose levels under fasting condition, and glycosylated hemoglobin levels (%) and TyG index (insulin resistance) after the whole experimental period (80 days) of the different experimental groups.

Experimental Group	Initial Glucose (mmol/L Blood)	Final Glucose (mmol/L Blood)	Glycosylated Hemoglobin (%)	TyG Index
ND	4.81 ± 0.62 ^c^	5.47 ± 0.57 ^c^	7.49 ± 0.87 ^b^	8.43 ± 0.19 ^c^
DC	26.41 ± 3.33 ^b^	28.08 ± 2.69 ^a^	14.92 ± 1.76 ^a^	11.02 ± 0.14 ^a^
D200	34.33 ± 2.66 ^a^	22.24 ± 3.27 ^bc^*	8.05 ± 0.87 ^b^	10.49 ± 0.35 ^b^
D400	32.29 ± 2.24 ^a^	18.85 ± 6.24 ^b^*	7.53 ± 1.12 ^b^	10.22 ± 0.27 ^b^
D600	31.92 ± 2.08 ^a^	15.93 ± 2.83 ^b^*	7.41 ± 1.02 ^b^	10.17 ± 0.34 ^b^

^a,b,c^ Different letters indicate significant differences between samples by Tukey post-hoc test (*p* ≤ 0.05), expressed as mean value ± S.D. * Indicate significant differences between initial and final glucose values by Tukey post-hoc test (*p* ≤ 0.05). ND = non-diabetic group; DC = control diabetic group; D200 = diabetic group treated with 200 mg/Kg; D400 = diabetic group treated with 400 mg/Kg; D600 = diabetic group treated with 600 mg/Kg.

**Table 5:** Biochemical parameters, and LDL/HDL index of the different experimental groups after the whole experimental period (80 days).

Experimental Group	CHO (mmol/L Blood)	TG (mmol/L Blood)	HDL (mmol/L Blood)	VLDL (mmol/L Blood)	LDL (mmol/L Blood)	LDL/HDL Index
ND	2.80 ± 0.25 ^bc^	1.07 ± 0.19 ^c^	1.30 ± 0.10 ^b^	0.21 ± 0.04 ^c^	1.29 ± 0.24 ^a^	1.00 ± 0.19 ^a^
DC	3.41 ± 0.29 ^a^	2.74 ± 0.15 ^a^	1.84 ± 0.15 ^a^	0.55 ± 0.03 ^a^	1.01 ± 0.31 ^ab^	0.57 ± 0.21 ^ab^
D200	2.87 ± 0.10 ^b^	2.13 ± 0.76 ^ab^	1.69 ± 0.33 ^a^	0.45 ± 0.15 ^ab^	0.75 ± 0.35 ^bc^	0.50 ± 0.38 ^b^
D400	2.43 ± 0.25 ^cd^	1.86 ± 0.39 ^b^	1.52 ± 0.30 ^ab^	0.38 ± 0.08 ^b^	0.48 ± 0.26 ^c^	0.32 ± 0.21 ^b^
D600	2.14 ± 0.24 ^d^	1.78 ± 0.35 ^bc^	1.50 ± 0.11 ^ab^	0.35 ± 0.08 ^bc^	0.28 ± 0.26 ^c^	0.19 ± 0.18 ^b^

^a,b,c,d^ Different letters indicate significant differences between samples by Tukey post-hoc test (*p* ≤ 0.05), expressed as mean value ± S.D. CHO = total cholesterol; TG = total triglycerides; HDL = total high density lipoproteins; VLDL = total very low density lipoproteins; LDL = total low density lipoproteins. ND = non-diabetic group; DC = control diabetic group; D200 = diabetic group treated with 200 mg/Kg; D400 = diabetic group treated with 400 mg/Kg; D600 = diabetic group treated with 600 mg/Kg.

## Data Availability

Data is unavailable due to privacy restrictions.

## Supplementary Materials

The following supporting information can be downloaded at: https://www.mdpi.com/article/10.3390/antiox13081010/s1, Figure S1. Phenolic compounds identification in *Hibiscus sabdariffa* extract through HPLC-DAD-Uv (chromatogram) for phenolic acids (a) and flavonoids (b).

## References

[1] Liu Y., Wang Q., Wu K., Sun Z., Tang Z., Li X., Zhang B. (2023). Anthocyanins’ effects on diabetes mellitus and islet transplantation. Crit. Rev. Food Sci. Nutr..

[2] Jamrozik D., Borymska W., Kaczmarczyk-Żebrowska I. (2022). *Hibiscus sabdariffa* in Diabetes Prevention and Treatment—Does It Work? An Evidence-Based Review. Food.

[3] Golovinskaia O., Wang C.K. (2023). The hypoglycemic potential of phenolics from functional foods and their mechanisms. Food Sci. Hum. Wellness.

[4] Upadhyay T.K., Das S., Mathur M., Alam M., Bhardwaj R., Joshi N., Sharangi A.B., Naeem M., Aftab T. (2024). Medicinal plants and their bioactive components with antidiabetic potentials. Book Antidiabetic Medicinal Plants. Applications and Opportunities.

[5] Magaña-Rodríguez O.R., Ortega-Pérez L.G., Ayala-Ruiz L.A., Piñon-Simental J.S., Gallegos-Torres O.F., Chavez P.R. (2023). Inhibitory Effects of Edible and Medicinal Plant Extracts on the Enzymatic Activity of Pancreatic Lipase. J. Mex. Chem. Soc..

[6] Li Z., Tian J., Cheng Z., Teng W., Zhang W., Bao Y., Li B. (2023). Hypoglycemic bioactivity of anthocyanins: A review on proposed targets and potential signaling pathways. Crit. Rev. Food Sci. Nutr..

[7] Swargiary A., Roy M.K., Mahmud S. (2023). Phenolic compounds as α-glucosidase inhibitors: A docking and molecular dynamics simulation study. J. Biomol. Struct. Dyn..

[8] Solverson P. (2020). Anthocyanin bioactivity in obesity and diabetes: The essential role of glucose transporters in the gut and periphery. Cell.

[9] Salau V.F., Erukainure O.L., Olofinsan K.O., Bharuth V., Ijomone O.M., Islam M.S. (2023). Ferulic acid improves glucose homeostasis by modulation of key diabetogenic activities and restoration of pancreatic architecture in diabetic rats. Fundam. Clin. Pharmacol..

[10] Martini D., Marino M., Venturi S., Tucci M., Klimis-Zacas D., Riso P., Del-Bo C. (2023). Blueberries and their bioactives in the modulation of oxidative stress, inflammation and cardio/vascular function markers: A systematic review of human intervention studies. J. Nutr. Biochem..

[11] Sun C., Zhao C., Guven E.C., Paoli P., Simal-Gandara J., Ramkumar K.M., Xiao J. (2020). Dietary polyphenols as antidiabetic agents: Advances and opportunities. Food Front..

[12] Naz R., Saqib F., Awadallah S., Wahid M., Latif M.F., Iqbal I., Mubarak M.S. (2023). Food Polyphenols and Type II Diabetes Mellitus: Pharmacology and Mechanisms. Molecules.

[13] García-Muñoz A.M., García-Guillén A.I., Victoria-Montesinos D., Abellán-Ruiz M.S., Alburquerque-González B., Cánovas F. (2023). Effect of the combination of *Hibiscus sabdariffa* in combination with other plant extracts in the prevention of metabolic syndrome: A Systematic Review and Meta-Analysis. Foods.

[14] Salami S.O., Afolayan A.J. (2020). Suitability of Roselle-*Hibiscus sabdariffa* L. as raw material for soft drink production. J. Food Qual..

[15] Amtaghri S., Qabouche A., Slaoui M., Eddouks M. (2024). A comprehensive overview of *Hibiscus rosa-sinensis* L.: Its ethnobotanical uses, phytochemistry, therapeutic uses, pharmacological activities, and toxicology. Endocr. Metab. Immune. Disord. Drug Targets.

[16] Montalvo-González E., Villagrán Z., González-Torres S., Iñiguez-Muñoz L.E., Isiordia-Espinoza M.A., Ruvalcaba-Gómez J.M., Anaya-Esparza L.M. (2022). Physiological effects and human health benefits of *Hibiscus sabdariffa*: A review of clinical trials. Pharmaceuticals.

[17] Cid-Ortega S., Guerrero-Beltrán J.A. (2015). Roselle calyces (*Hibiscus sabdariffa*), an alternative to the food and beverages industries: A review. J. Food Sci. Technol..

[18] Rasheed D.M., Porzel A., Frolov A., El Seedi H.R., Wessjohann L.A., Farag M.A. (2018). Comparative analysis of *Hibiscus sabdariffa* (roselle) hot and cold extracts in respect to their potential for α-glucosidase inhibition. Food Chem..

[19] Alegbe E.O., Teralı K., Olofinsan K.A., Surgun S., Ogbaga C.C., Ajiboye T.O. (2019). Antidiabetic activity-guided isolation of gallic and protocatechuic acids from *Hibiscus sabdariffa* calyxes. J. Food Biochem..

[20] Hapsari B.W., Manikharda-Setyaningsih W. (2021). Methodologies in the Analysis of Phenolic Compounds in Roselle (*Hibiscus sabdariffa* L.): Composition, Biological Activity, and Beneficial Effects on Human Health. Horticulturae.

[21] Nieto J.A., Santoyo S., Prodanov M., Reglero G., Jaime L. (2020). Valorisation of grape stems as a source of phenolic antioxidants by using a sustainable extraction methodology. Foods.

[22] Nieto J.A., Fernández-Jalao I., Siles-Sánchez M.D.L.N., Santoyo S., Jaime L. (2023). Implication of the Polymeric Phenolic Fraction and Matrix Effect on the Antioxidant Activity, Bioaccessibility, and Bioavailability of Grape Stem Extracts. Molecules.

[23] Soares L.A.L., Bassani V.L., González Ortega G., Petrovick P.R. (2003). Total Flavonoid Determination for the Quality Control of Aqueous Extractives from *Phyllanthus niruri* L.. Acta Farm. Bonaer..

[24] Prior R.L., Cao G., Martin A., Sofic E., McEwen J., O’Brien C., Mainland C.M. (1998). Antioxidant Capacity as Influenced by Total Phenolic and Anthocyanin Content, Maturity, and Variety of Vaccinium Species. J. Agric. Food Chem..

[25] Re R., Pellegrini N., Proteggente A., Pannala A., Yang M., Rice-Evans C. (1999). Antioxidant activity applying an improved ABTS radical cation decolorization assay. Free Radical Biol. Med..

[26] Brand-Williams W., Cuvelier M.E., Berset C.L.W.T. (1995). Use of a free radical method to evaluate antioxidant activity. LWT Food Sci. Technol..

[27] Xie Z., Huang J., Xu X., Jin Z. (2008). Antioxidant activity of peptides isolated from alfalfa leaf protein hydrolysate. Food Chem..

[28] Ademiluyi A.O., Oboh G. (2013). Aqueous extracts of Roselle (*Hibiscus sabdariffa* Linn.) varieties inhibit α-amylase and α-glucosidase activities in vitro. J. Med. Food.

[29] Zhou Z., Wang F., Ren X., Wang Y., Blanchard C. (2015). Resistant starch manipulated hyperglycemia/hyperlipidemia and related genes expression in diabetic rats. Int. J. Biol. Macromol..

[30] Kim W.K., Chung M.K., Kang N.E., Kim M.H., Park O.J. (2003). Effect of resistant starch from corn or rice on glucose control, colonic events, and blood lipid concentrations in streptozotocin-induced diabetic rats. J. Nutr. Biochem..

[31] Kumar V., Akinleye A.O., Makkar H.P., Angulo-Escalante M.A., Becker K. (2012). Growth performance and metabolic efficiency in *Nile tilapia* (*Oreochromis niloticus* L.) fed on a diet containing Jatropha platyphylla kernel meal as a protein source. J. Anim. Physiol. Anim..

[32] Suárez-Diéguez T., Nájera M.O., Galván M., Nieto J.A. (2023). Impact of a retrograded starch ingredient obtained from Negro Jamapa beans (*Phaseolus vulgaris* L. Negro Jamapa) on glucose metabolism and oxidative stress in induced diabetic lab rats model. Int. J. Biol. Macromol..

[33] Unger G., Benozzi S.F., Perruzza F., Pennacchiotti G.L. (2014). Índice triglicéridos y glucosa: Un indicador útil de insulinorresistencia. Endocrinol Nutr..

[34] Peredo Pozos G.I., Ruiz-López M.A., Zamora Natera J.F., Alvarez Moya C., Barrientos-Ramirez L., Reynoso-Silva M., Vargas-Radillo J.J. (2020). Antioxidant capacity and antigenotoxic effect of *Hibiscus sabdariffa* L. extracts obtained with ultrasound-assisted extraction process. Appl. Sci..

[35] Alara O.R., Abdurahman N.H., Obanijesu E.O., Alara J.A., Abdul-Mudalip S.K. (2020). Extract-rich in flavonoids from *Hibiscus sabdariffa* calyces: Optimizing microwave-assisted extraction method and characterization through LC-Q-TOF-MS analysis. J. Food Eng..

[36] Mercado-Mercado G., Blancas-Benitez F.J., Velderrain-Rodríguez G.R., Montalvo-González E., González-Aguilar G.A., Alvarez-Parrilla E., Sáyago-Ayerdi S.G. (2015). Bioaccessibility of polyphenols released and associated to dietary fibre in calyces and decoction residues of Roselle (*Hibiscus sabdariffa* L.). J. Funct. Foods..

[37] Diez-Echave P., Vezza T., Rodriguez-Nogales A., Ruiz-Malagón A.J., Hidalgo-Garcia L., Garrido-Mesa J., Molina-Tijeras J.A., Romero M., Robles-Vera I., Pimentel-Moral S. (2020). The prebiotic properties of *Hibiscus sabdariffa* extract contribute to the beneficial effects in diet-induced obesity in mice. Food Res. Inter..

[38] Al-Yousef H.M., Hassan W.H., Abdelaziz S., Amina M., Adel R., El-Sayed M.A. (2020). UPLC-ESI-MS/MS Profile and Antioxidant, Cytotoxic, Antidiabetic, and Antiobesity Activities of the Aqueous Extracts of Three Different Hibiscus Species. J. Chem..

[39] Maciel L.G., do Carmo M.A.V., Azevedo L., Daguer H., Molognoni L., de Almeida M.M., Granato D., Rosso N.D. (2018). *Hibiscus sabdariffa* anthocyanins-rich extract: Chemical stability, in vitro antioxidant and antiproliferative activities. Food Chem. Toxicol..

[40] Sachdewa A., Khemani L.D. (2003). Effect of Hibiscus rosa sinensis Linn. ethanol flower extract on blood glucose and lipid profile in streptozotocin induced diabetes in rats. J. Ethnopharmacol..

[41] Adeyemi D.O., Adewole O.S. (2019). *Hibiscus sabdariffa* renews pancreatic β-cells in experimental type 1 diabetic model rats. Morphologie.

[42] Venkatesh S., Thilagavathi J. (2008). Anti-diabetic activity of flowers of Hibiscus rosasinensis. Fitoterapia.

[43] Adeyemi D.O., Ukwenya V.O., Obuotor E.M., Adewole S.O. (2014). Anti-hepatotoxic activities of *Hibiscus sabdariffa* L. in animal model of streptozotocin diabetes-induced liver damage. BMC Complement. Altern. Med..

[44] Rodríguez-Pérez C., Segura-Carretero A., del Mar Contreras M. (2019). Phenolic compounds as natural and multifunctional anti-obesity agents: A review. Crit. Rev. Food Sci. Nutr..

