# Supplementary material for: Modulation of the Hyperglycemia Condition in Diabetic Lab Rats with Extracts of the Creole Jamaica Flower (Hibiscus sabdariffa L.) from the Morelia Region (Mexico)
Source: Antioxidants (Basel). 2024 Aug 19;13(8):1010. doi: 10.3390/antiox13081010 (PMC11352102; doi:10.3390/antiox13081010)
Supplement: Supplementary file 1 [file antioxidants-13-01010-s001.zip › antioxidants-3116452-supplementary.pdf]

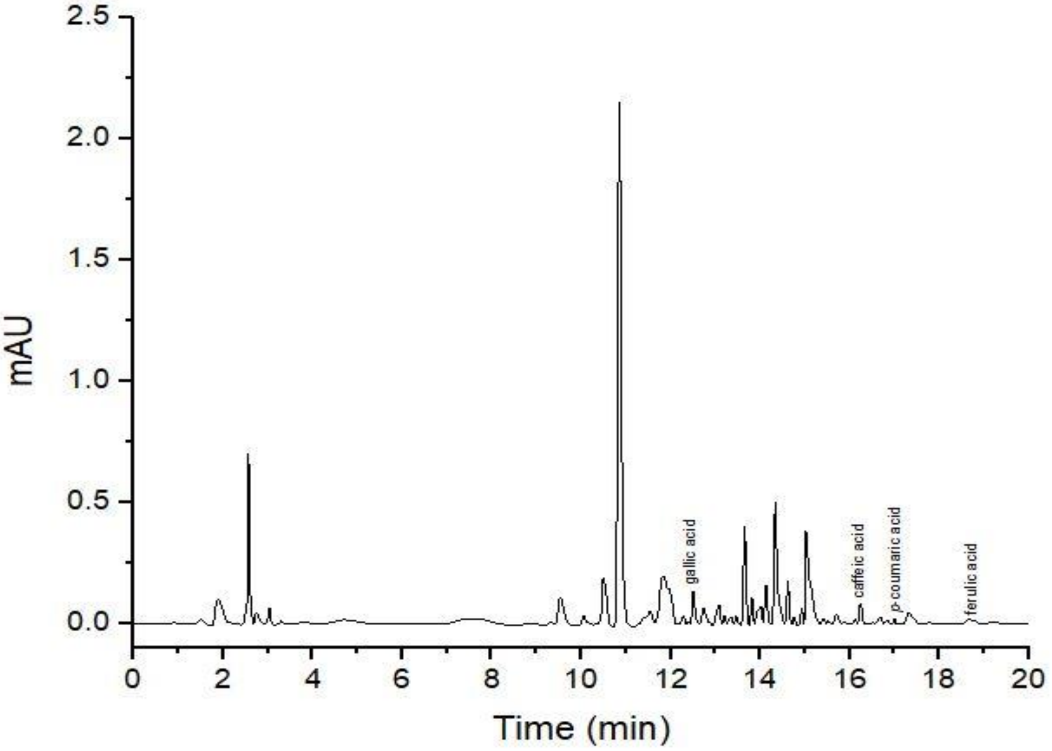

(**a**)

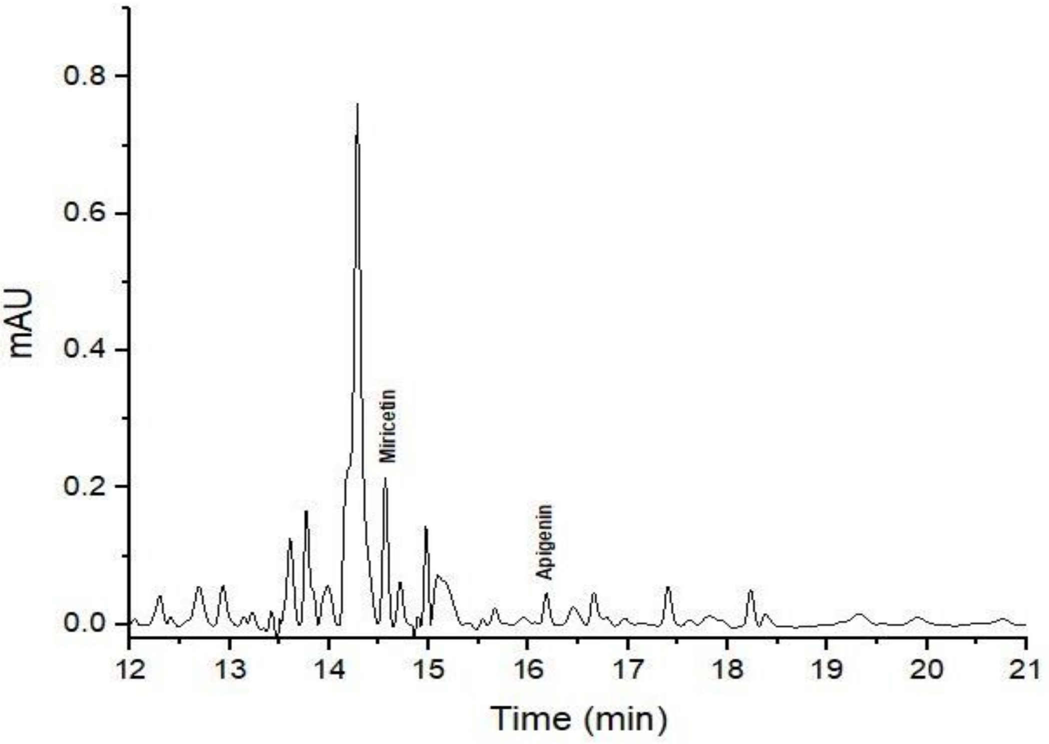

(**b**)

Figure S1. Phenolic compounds identification in *Hibiscus sabdariffa* extract through HPLC-DAD-Uv (chromatogram) for phenolic acids (a) and flavonoids (b)
